# Construction of an artificial cell membrane anchor using DARC as a fitting for artificial extracellular functionalities of eukaryotic cells

**DOI:** 10.1186/1477-3155-10-1

**Published:** 2012-01-05

**Authors:** Markus von Nickisch-Rosenegk, Till Teschke, Frank F Bier

**Affiliations:** 1 Fraunhofer IBMT, Am Muehlenberg 13, 14476 Potsdam, Germany

**Keywords:** Duffy, DARC, artificial cell membrane anchor, artificial cell membrane functionalities, recombinant functional membrane fusion protein

## Abstract

The need to functionalize cell membranes in a directed way for specific applications as single cell arrays or to force close cell-to-cell contact for artificial intercellular interaction and/or induction concerning stem cell manipulation or in general to have a tool for membrane and cell surface-associated processes, we envisaged a neutral inactive membrane anchor for extracellular entities to facillitate the above mentioned functionalities.

The silent Duffy antigen/receptor for chemokines (DARC) is a receptor-like membrane protein of erythrocytes and mediates no cell transduction not at least regarding a missing or truncated G-loop and therefore it seemed to be the candidate for our cell membrane anchor.

We isolated the genetic information of DARC from human genomic DNA and cloned it in a mammalian cell line as a fusion protein via a suitable plasmid vector.

In this report we demonstrate that the human plasma membrane protein DARC can be used as an artificial anchor molecule in cell surface engineering applications. We constructed the fusion protein SNAP-tag-DARC, consisting of DARC and the self-labeling protein tag SNAP-tag^® ^(Covalys). The SNAP-tag® served as an example for a molecular-technological developed protein that is artificially attached to the extracellular side of the plasma membrane through our DARC-anchor. SnapTag should serve as an example for any extracellular entity and was easy to detect by a commercial detection system. The synthesis of SNAP-tag-DARC, its correct incorporation into the cell membrane and the functionality of the SNAP-tag® were verified by RT-PCR, Western blotting and confocal fluorescence microscopy and showed the desired functionality as an membrane anchor for an extracellular application entity.

## Background

Currently desired manipulations of eukaryotic cells comprise the specific modification of their extracellular surface, in particular the cell membrane. Cell membranes can be engineered by attaching new molecules or by modifying or removing their natural components. Of particular interest are proteins, peptides, oligosaccharides and small organic molecules. In combination with other technologies, such modifications of the cell membrane shall enable a range of applications, as cell adhesion in general, single cell arrays, the sorting of cells on surfaces, the manipulation of migration and differentiation in cell cultures, the connection of naturally incompatible cell types, the mutual influence of cells staying in closed contact, artificial epitopes, the artificial fusion of cells, the engineering of antigen presenting cells, the reduction of graft rejection and the development of hybrid materials and systems [1-3].

The presentation of new molecules on the cell surface can be achieved by attaching them as a fusion partner protein to a transmembrane protein through conventional gene transfer.

In this work, we examined whether the human plasma membrane protein DARC [4] can act as such a nanobiotechnological polypeptide anchor fused with a functional peptide of interest.

DARC was chosen because it is functionally redundant and not known to initiate intracellular signal transduction [5]. It is a glycoprotein with the predicted topology of a G protein-coupled receptor. Its major isoform theoretically consists of 336 amino acids. DARC carries the antigenic determinants of the human blood group system Duffy and is an erythrocyte co-receptor for the merozoites of the human malaria parasite *Plasmodium vivax *[6,7]. In addition, DARC is a silent chemokine receptor that binds several chemokines, but shows no G protein-coupled signal transduction. DARC lacks the typical DRY-motif on the second intracellular loop, which is an essential requirement for signal transduction of all residual polypeptides of the GPCR family [8].

DARC is present on erythrocytes and certain endothelial cells of terminal vessels, but also on some epithelial cells. It is produced in special vessels of kidney, lung, thyroid and spleen, especially along postcapillary venules. DARC plays a role in metastasis suppression and inflammation as extracellular functionalities but seemingly missing the intracellular ones as described above [9-11]. Therefore it is the best candidate for an transfectable artificial cell membrane anchor, which promise to behave inert within target cells.

The SNAP-tag® was chosen to exemplify a protein attached to DARC that protrudes into the extracellular space as an artificial functionality. Additionally, SNAP-tag® was used as a method for detecting the intended SNAP-tag-DARC fusion proteins on the surface of transfected cells. The SNAP-tag® is a self-labeling protein tag derived from the human O^6^-alkylguanine-DNA alkyltransferase (AGT) [12]. SNAP-tag® can establish a covalent bond between the benzyl group of O^6^-benzylguanine and a cysteine in its active center, releasing guanine. By fusing the SNAP-tag® to a protein of interest and using a substrate carrying a fluorophore on its benzyl group, one can specifically and covalently label the protein of interest. Under physiological conditions, the reaction is irreversible. The SNAP-tag® was chosen because it allows cell surface labeling along with cell impermeable substrates. Intracellular SNAP-tag-DARC and endogenous AGT are not labeled.

## Results and discussion

In order to create an anchor which is located within the cell membrane of eukaryotic cells and is able to provide extracellular functionalities, we chose the 7-trans-membrane protein DARC. DARC is a silent receptor regarding the intracellular transduction cascade and therefore promises a suitable building block as a membrane anchor for an extracellular and artificial receptor for controlled applications with specific ligands. The chosen DARC was amplified from human genomic DNA by PCR and the synthesis products were ligated in an expression plasmid. The SNAP-tag® ligated to the DARC anchor served as an example for a possible extracellular functionality. The correct sequences and the functional ligation of the gene fragments were verified by commercial sequencing.

**Figure 1:**
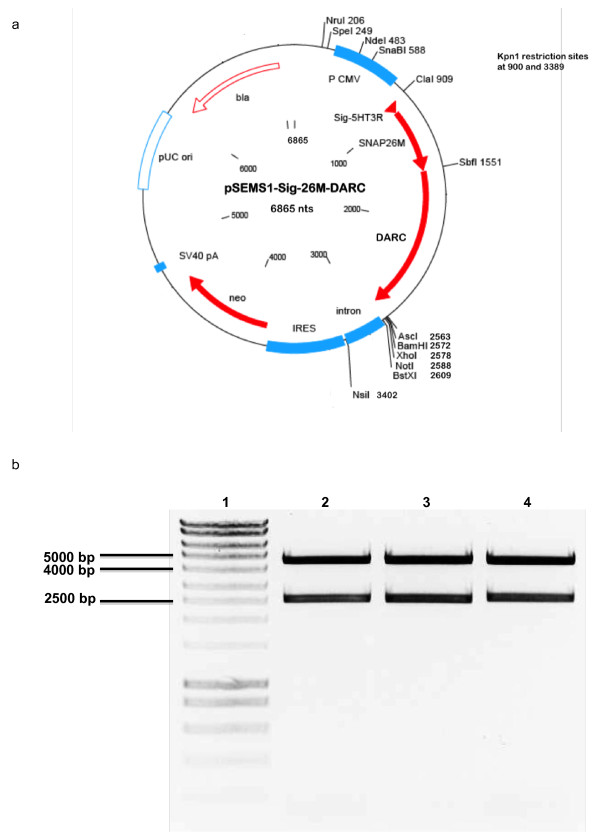
**Final construct of SNAP-Tag-DARC**. In 1a the complete vector map of the final construct enabling the SNAP-Tag-DARC fusion protein is shown. 1b represents an agarose gel of the related plasmid after digestion with KpnI, which has two restriction sites flanking the complete SNAP-Tag-DARC construct lane 1 = size marker, lane 2-4 = two bands of the digested plasmid.

In Figure 1 the final construct of SNAP-tag-DARC is shown in the plasmid pSEMS1-Sig-26 as a vector-map and in an agarose gel where the plasmid is digested by restriction enzymes. A suitable enzyme, which cuts the plasmid two times could be identified from the plasmid sequence shown in additional file 1. The KpnI digestion generates two fragments of around 4500 bp and 2500 bp. The latter represents the genes of the fusion protein SNAP-tag-DARC plus some basepairs of the vector backbone caused by the KpnI restriction sites (see 1b)

**Figure 2:**
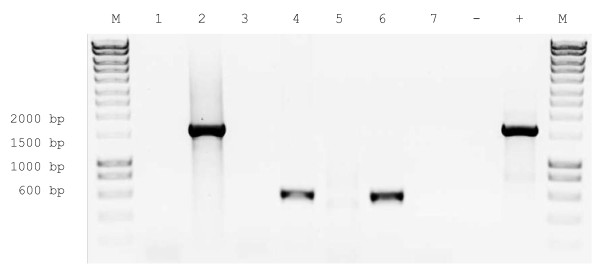
**Agarose gel of RT-PCR of transfected cells (lanes 1 - 4) and untransfected cells (Lanes 5 - 7)**. Transcripts of the SNAP-tag-DARC fusion gene were detected (lane 2). 1) no RT control with SNAP-tag-DARC primers, 3) no RT control with ATPase primers, 4) RT positive control with ATPase primers (housekeeping gene), 5) RT control with SNAP-tag-DARC primers, 6) RT positive control with ATPase primers (housekeeping gene), 7) no RT control with ATPase primers, -) PCR control without primers, +) PCR control with SNAP-tag-DARC primers and total DNA from transfected cells, M) size marker.

In order to examine whether the SNAP-tag-DARC fusion gene was properly transcribed in transfected cells, RT-PCR was performed. In transfected cells the transcripts of the SNAP-tag-DARC fusion gene were detected in the correct size, which could be observed in the first four lanes of an agarose gel in Figure 2. The transcript of the SNAP-tag-DARC fusion gene could be visualized as well as in the transfected cells used positive control (ATPase). Control reactions of the transfected cells containing no reverse transcriptase (RT) proved that the signals resulted from cDNA and not from plasmid DNA used to transfect the cells.

The transcript of a housekeeping gene for ATPase served as a positive control for the RT-PCR reaction even in the reactions of the untransfected cells (lane 6), which are visualized in the lanes five to seven of Figure 2.

Subsequent restriction of the RT-PCR products with SbfI showed that they were hydrolyzed in the sizes as expected for SNAP-tag-DARC DNA (data not shown).

**Figure 3:**
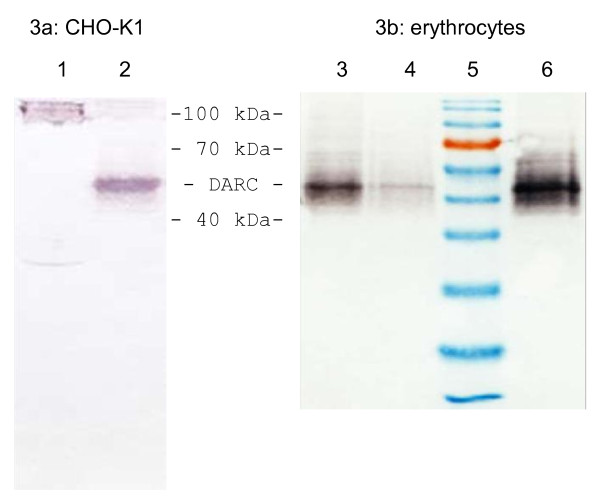
**Western Blot of DARC in transfected cells and erythrocytes**. In 3a of total membrane fraction of CHO-K1 cells (lane 1) and solubilized membrane fraction of erythrocytes (lane 2) co-incubated with antibodies against the DARC- and the Transferrin receptor. Lane 1 shows the expected band of Transferrin of around 100 kDa. In figure 3b different protein loads are shown: lane 3 carries 10 μg of solubilized erythrocyte membrane; lane 4 = 5 μg; lane 6 = 15 μg; lane 5 = molecular weight marker.

In order to examine whether SNAP-tag-DARC was synthesized in the transfected cells, Western Blotting was performed. SNAP-tag-DARC was expected to have a molecular mass of 58 to 69 kDa, depending on the extent of DARC's glycosylation (with 69 kDa corresponding to full glycosylation, and 58 kDa corresponding to no glycosylation). To elucidate possible cross-reactivity we blotted the complete membrane fraction of the used CHO-K1 cells and of erythrocytes and incubated the blots with anti-DARC and anti-Transferrin antibodies shown in Figure 3a. In contrast to the Transferrin-positive control, no signal of DARC is visible in the CHO-K1 membrane fraction. For comparison different amounts of extracts from erythrocytes are shown in Figure 3b.

In order to examine whether SNAP-tag-DARC is localized to the plasma membrane and was incorporated with the SNAP-tag® protruding into the extracellular space, cells were treated with the cell impermeable SNAP-tag® substrate BG-488. The strategy of using the SNAP-tag® with a cell impermeable substrate to prove correct localization of the fusion protein has the advantage that endogenous SNAP-tag-DARC will not be labeled. A recent study has shown that different SNAP-tag fusion proteins, despite possessing a reactive cysteine, can be labeled with fluoresceine and the transport of the protein could be tracked through the membrane and to the extracellular side of the plasma membrane [13].

As a control for the correct localization of a recombinant membrane receptor fusion protein, we transfected CHO-K1 cells with an other G-protein coupled receptor gene. The Adrenergic Receptor Beta 2 (ADRB2) was cloned in analogy to DARC and co-expressed with the SNAP-tag.

**Figure 4:**
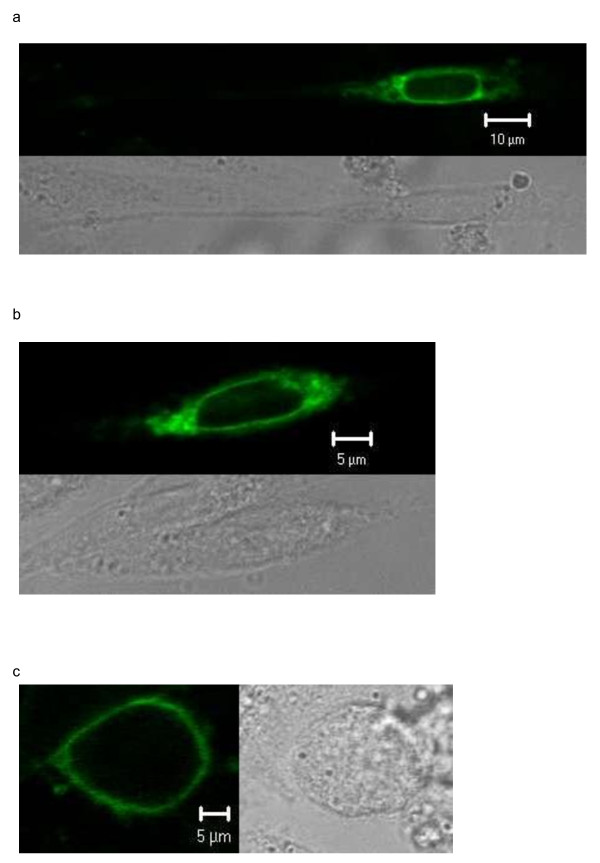
**Transiently transfected CHO-K1 cells expressing the SNAP-tag-DARC fusion gene in the Membrane**. Cells were labeled with the cell impermeable SNAP-tag® substrate BG-488. Top of 4a and 4b; left of 4c; Fluorescence channel, optical slice < 1.2 μm. Bottom of 4a and 4b and right of 4c: Same section in transmitted light.

**Figure 5:**
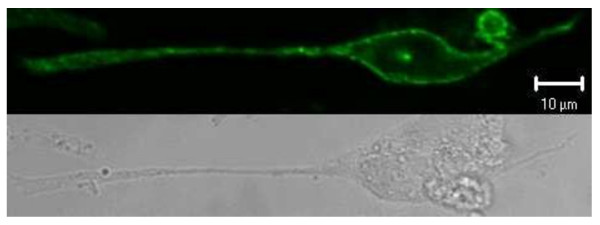
**Transiently transfected CHO-K1 cells expressing the cell surface localization control SNAP-tag-ADRB2 receptor gene in the cell membrane**. Cells were labeled with the cell impermeable SNAP-tag® substrate BG-488. Top; Fluorescence channel, optical slice < 1.5 μm. Bottom: Same section in transmitted light.

The labeling of transfected SNAP-tag-DARC cells with BG-488 was successful. Confocal micrographs of transfected cells showed homogeneous surface labeling (Figure 4). The fluorescing structures were congruent with the rims of the cells as seen in transmitted light. Fluorescence signals corresponded even well with those of the cell surface localization control, SNAP-tag-ADRB2-receptor (Figure 5). These results were reproducible in three independent transfection and labeling experiments.

For control of autofluorescence of transfected cells or unspecific binding of BG-488 to untransfected cells (the letter were incubated with the BG-488 substrate), fluorescence microscopy has been performed. No fluorescence could be detected

## Methods

### Preparation of DARC gene

Human genomic DNA was amplified with the primers (DARC-SbfI-forward: gggctgggtcctgcaggtatggcctcctctgggtatgtcc and DARC-XhoI-reverse: gtgtgtcactcgaggctaggatttgcttccaagggtgtcc) spanning arround 1000 bp and constructed with homology of the 3'- and the 5'-end of the DARC exon (Genbank: AM887935) and for cloning with suitable restriction sites enabling in-frame ligation in the appropriate expression vector.

### Construction of the SNAP-tag-DARC expression plasmid

For the construction of SNAP-tag-DARC and a correct localization to the cell surface, DARC was fused to the C terminus of a SNAP-tag® equipped with a suitable signal peptide. For this purpose, the human gene *DARC *(exon 2 encoding the minor isoform of 338 amino acids) was amplified by PCR and cloned into the *Sbf*I and *Xho*I sites of the vector pSEMS1-Sig-26m (Covalys).

### Cell culture and transfection

CHO-K1 cells from the German Collection of Microorganisms and Cell Cultures were grown in Ham's F-12 supplemented with 10% FCS (Biochrom). Cells were transiently transfected with the recombinant vector pSEMS1-Sig-26m-DARC using FuGENE HD (Roche).

### Sequencing of the cloned vector construct

Subsequent sequencing from plasmid prep of transfected cells was accomplished commercially by Agowa GmbH to confirm the construct.

### RT-PCR

40 hours following transfection, total RNA was extracted from the cells and treated with DNase I. After DNase inactivation, RNA was reverse-transcribed and cDNA was amplified by PCR.

### Western Blot

72 hours following transfection, total cellular membrane proteins were extracted from the cells. Proteins were analyzed by Western Blot using a monoclonal antibody to human blood group Fy6 (Becton Dickinson) and a monoclonal antibody to human transferrin receptor (Invitrogen) at final concentrations of 0,50 μg/ml and 0,17 μg/ml, respectively. For protein denaturation, no reducing agent was used.

### Fluorescence labeling

Cells were grown on chambered coverglass slides. 24 to 48 hours after transfection, the cell impermeable SNAP-tag® substrate BG-488 (Covalys) was added to the medium to a final concentration of 10 μM. Cells were incubated for 10 minutes in the incubator, washed with ice cold medium and fixed with 4% paraformaldehyde.

Laser-scanning confocal micrographs were recorded using a 488 nm laser line and appropriate filter sets on a Zeiss LSM 510 microscope with an x40 oil objective (Zeiss).

## Conclusion

Our experiments show that DARC can serve as a functional membrane anchor, which allows an extracellular fusion protein to react with an offered ligand in living eukaryotic cells. DARC was correctly integrated within the cell membrane presenting the aminoterminally fused enzyme protein to the extracellular space.

These results provide us a tool to functionalize cell membranes with well-defined entities. Further experiments should show the influence of artificially closed attached ligands to relevant target cells to examine and elucidate functions and influence and to understand protein to protein interactions.

## Competing interests

The authors declare that they have no competing interests.

## Authors' contributions

MvNR has createtd the basic idea and made substantial contributions to conception and design and wrote 50% of the manuscript. TT performed the experiments, made substantial contributions to the analysis and interpretation of the data and wrote 50% of the manuscript. FB has been involved in revising the manuscript critically for important intellectual content and has given final approval of the version to be published. All authors have read and approved the final manuscript.

## Supplementary Material

Additional file 1 **Complete sequence of the pSEMS1-Sig-26m-DARC vector: DNA sequence of the Covalys SNAP-tag-DARC plasmid containing the genes for the fusion protein of SNAP-tag and DARC**. Click here for file
