# Supplementary material for: Construction of an artificial cell membrane anchor using DARC as a fitting for artificial extracellular functionalities of eukaryotic cells
Source: J Nanobiotechnology. 2012 Jan 5;10:1. doi: 10.1186/1477-3155-10-1 (PMC3271035; doi:10.1186/1477-3155-10-1)
Supplement: Additional file 1 — Complete sequence of the pSEMS1-Sig-26m-DARC vector: DNA sequence of the Covalys SNAP-tag-DARC plasmid containing the genes for the fusion protein of SNAP-tag and DARC. [file 1477-3155-10-1-S1.PDF]

Sequence of pSEMS1-Sig-26M-DARC, 6865 nts

GACGGATCGGGAGATCTCCCGATCCCCTATGGTCGACTCTCAGTACAATCTGCTCTGATGCCGCATAGTTAAGCCAGTAT
CTGCTCCCTGCTTGTGTGTTGGAGGTCGCTGAGTAGTGCGCGAGCAAAATTTAAGCTACAACAAGGCAAGGCTTGACCGA
CAATTGCATGAAGAATCTGCTTAGGGTTAGGCGTTTTGCGCTGCTTCGCGATGTACGGGCCAGATATACGCGTTGACATT
GATTATTGACTAGTTATTAATAGTAATCAATTACGGGGTCATTAGTTCATAGCCCATATATGGAGTTCCGCGTTACATAA
CTTACGGTAAATGGCCCGCCTGGCTGACCGCCCAACGACCCCCGCCCATTGACGTCAATAATGACGTATGTTCCCATAGT
AACGCCAATAGGGACTTTCCATTGACGTCAATGGGTGGACTATTTACGGTAAACTGCCCACTTGGCAGTACATCAAGTGT
ATCATATGCCAAGTACGCCCCCTATTGACGTCAATGACGGTAAATGGCCCGCCTGGCATTATGCCCAGTACATGACCTTA
TGGGACTTTCCTACTTGGCAGTACATCTACGTATTAGTCATCGCTATTACCATGGTGATGCGGTTTTGGCAGTACATCAA
TGGGCGTGGATAGCGGTTTGACTCACGGGGATTTCCAAGTCTCCACCCCATTGACGTCAATGGGAGTTTGTTTTGGCACC
AAAATCAACGGGACTTTCCAAAATGTCGTAACAACTCCGCCCCATTGACGCAAATGGGCGGTAGGCGTGTACGGTGGGAG
GTCTATATAAGCAGAGCTCTCTGGCTAACTAGAGAACCCACTGCTTACTGGCTTATCGAAATTAATACGACTCACTATAG
GGAGACCCAAGCTTGGTACCGAGCTCGGATCGATATCACCATGCGGCTCTGCATCCCGCAGGTGCTGTTGGCCTTGTTCC
TTTCCATGCTGACAGGGCCGGGAGAAGGCAGCCGGAAGCTTACCATGGACAAAGACTGCGAAATGAAGCGCACCACCCTG
GATAGCCCTCTGGGCAAGCTGGAACTGTCTGGGTGCGAACAGGGCCTGCACGAGATCAAGCTGCTGGGCAAAGGAACATC
TGCCGCCGACGCCGTGGAAGTGCCTGCCCCAGCCGCCGTGCTGGGCGGACCAGAGCCACTGATGCAGGCCACCGCCTGGC
TCAACGCCTACTTTCACCAGCCTGAGGCCATCGAGGAGTTCCCTGTGCCAGCCCTGCACCACCCAGTGTTCCAGCAGGAG
AGCTTTACCCGCCAGGTGCTGTGGAAACTGCTGAAAGTGGTGAAGTTCGGAGAGGTCATCAGCTACCAGCAGCTGGCCGC
CCTGGCCGGCAATCCCGCCGCCACCGCCGCCGTGAAAACCGCCCTGAGCGGAAATCCCGTGCCCATTCTGATCCCCTGCC
ACCGGGTGGTGTCTAGCTCTGGCGCCGTGGGGGGCTACGAGGGCGGGCTCGCCGTGAAAGAGTGGCTGCTGGCCCACGAG
GGCCACAGACTGGGCAAGCCTGGGCTGGGTCCTGCAGGTATGGCCTCCTCTGGGTATGTCCTCCAGGCGGAGCTCTCCCC
CTCAACTGAGAACTCAAGTCAGCTGGACTTCGAAGATGTATGGAATTCTTCCTATGGTGTGAATGATTCCTTCCCAGATG
GAGACTATGATGCCAACCTGGAAGCAGCTGCCCCCTGCCACTCCTGTAACCTGCTGGATGACTCTGCACTGCCCTTCTTC
ATCCTCACCAGTGTCCTGGGTATCCTAGCTAGCAGCACTGTCCTCTTCATGCTTTTCAGACCTCTCTTCTGCTGGCAGCT
CTGCCCTGGCTGGCCTGTCCTGACACAGCTGGCTGTGGGCAGTGCCCTCTTCAGCATTGTGGTGCCCGTCTTGGCCCCAG
GGCTAGGTAGCACTCGCAGCTCTGCCCTGTGTAGCCTGGGCTACTGTGTCTGGTATGGCTCAGCCTTTGCCCAGGCTTTG
CTGCTAGGGTGCCATGCCTCCCTGGGCCACAGACTGGGTGCAGGCCAGGTCCCAGGCCTCACCCTGGGGCTCACTGTGGG
AATTTGGGGAGTGGCTGCCCTACTGACACTGCCTGTCACCCTGGCCAGTGGTGCTTCTGGTGGACTCTGCACCCTGATAT
ACAGCACGGAGCTGAAGGCTTTGCAGGCCACACACACTGTAGCCTGTCTTGCCATCTTTGTCTTGTTGCCATTGGGTTTG
TTTGGAGCCAAGGGGCTGAAGAAGGCATTGGGTATGGGGCCAGGCCCCTGGATGAATATCCTGTGGGCCTGGTTTATTTT
CTGGTGGCCTCATGGGGTGGTTCTAGGACTGGATTTCCTGGTGAGGTCCAAGCTGTTGCTGTTGTCAACATGTCTGGCCC
AGCAGGCTCTGGACCTGCTGCTGAACCTGGCAGAAGCCCTGGCAATTTTGCACTGTGTGGCTACGCCCCTGCTCCTCGCC
CTATTCTGCCACCAGGCCACCCGCACCCTCTTGCCCTCTCTGCCCCTCCCTGAAGGATGGTCTTCTCATCTGGACACCCT
TGGAAGCAAATCCTAGCCTCGAGTGAGGCGGCCGCATAGATAACTGATCCAGTGTGCTGGAATTAATTCGCTGTCTGCGA
GGGCCAGCTGTTGGGGTGAGTACTCCCTCTCAAAAGCGGGCATGACTTCTGCGCTAAGATTGTCAGTTTCCAAAAACGAG
GAGGATTTGATATTCACCTGGCCCGCGGTGATGCCTTTGAGGGTGGCCGCGTCCATCTGGTCAGAAAAGACAATCTTTTT
GTTGTCAAGCTTGAGGTGTGGCAGGCTTGAGATCTGGCCATACACTTGAGTGACAATGACATCCACTTTGCCTTTCTCTC
CACAGGTGTCCACTCCCAGGTCCAACTGCAGGTCGAGCATGCATCTAGGGCGGCCAATTCCGCCCCTCTCCCTCCCCCCC
CCCTAACGTTACTGGCCGAAGCCGCTTGGAATAAGGCCGGTGTGCGTTTGTCTATATGTTATTTTCCACCATATTGCCGT
CTTTTGGCAATGTGAGGGCCCGGAAACCTGGCCCTGTCTTCTTGACGAGCATTCCTAGGGGTCTTTCCCCTCTCGCCAAA
GGAATGCAAGGTCTGTTGAATGTCGTGAAGGAAGCAGTTCCTCTGGAAGCTTCTTGAAGACAAACAACGTCTGTAGCGAC
CCTTTGCAGGCAGCGGAACCCCCCACCTGGCGACAGGTGCCTCTGCGGCCAAAAGCCACGTGTATAAGATACACCTGCAA
AGGCGGCACAACCCCAGTGCCACGTTGTGAGTTGGATAGTTGTGGAAAGAGTCAAATGGCTCTCCTCAAGCGTATTCAAC
AAGGGGCTGAAGGATGCCCAGAAGGTACCCCATTGTATGGGATCTGATCTGGGGCCTCGGTGCACATGCTTTACATGTGT
TTAGTCGAGGTTAAAAAAACGTCTAGGCCCCCCGAACCACGGGGACGTGGTTTTCCTTTGAAAAACACGATGATAAGCTT
GCCACAACCCGGGATAATTCCTGCAGCCAATATGGGATCGGCCATTGAACAAGATGGATTGCACGCAGGTTCTCCGGCCG
CTTGGGTGGAGAGGCTATTCGGCTATGACTGGGCACAACAGACAATCGGCTGCTCTGATGCCGCCGTGTTCCGGCTGTCA
GCGCAGGGGCGCCCGGTTCTTTTTGTCAAGACCGACCTGTCCGGTGCCCTGAATGAACTGCAGGACGAGGCAGCGCGGCT
ATCGTGGCTGGCCACGACGGGCGTTCCTTGCGCAGCTGTGCTCGACGTTGTCACTGAAGCGGGAAGGGACTGGCTGCTAT
TGGGCGAAGTGCCGGGGCAGGATCTCCTGTCATCTCACCTTGCTCCTGCCGAGAAAGTATCCATCATGGCTGATGCAATG
CGGCGGCTGCATACGCTTGATCCGGCTACCTGCCCATTCGACCACCAAGCGAAACATCGCATCGAGCGAGCACGTACTCG
GATGGAAGCCGGTCTTGTCGATCAGGATGATCTGGACGAAGAGCATCAGGGGCTCGCGCCAGCCGAACTGTTCGCCAGGC
TCAAGGCGCGCATGCCCGACGGCGATGATCTCGTCGTGACCCATGGCGATGCCTGCTTGCCGAATATCATGGTGGAAAAT
GGCCGCTTTTCTGGATTCATCGACTGTGGCCGGCTGGGTGTGGCGGACCGCTATCAGGACATAGCGTTGGCTACCCGTGA
TATTGCTGAAGAGCTTGGCGGCGAATGGGCTGACCGCTTCCTCGTGCTTTACGGTATCGCCGCTCCCGATTCGCAGCGCA
TCGCCTTCTATCGCCTTCTTGACGAGTTCTTCTGAGGGGATCAATTCTCTAGATAACTGATCATAATCAGCCATACCACA
TTTGTAGAGGTTTTACTTGCTTTAAAAAACCTCCCACACCTCCCCCTGAACCTGAAACATAAAATGAATGCAATTGTTGT
TGTTAACTTGTTTATTGCAGCTTATAATGGTTACAAATAAAGCAATAGCATCACAAATTTCACAAATAAAGCATTTTTTT
CACTGCATTCTAGTTGTGGTTTGTCCAAACTCATCAATGTATCTTAACGCGTCGAGTGCATTCTAGTTGTGGTTTGTCCA
AACTCATCAATGTATCTTATCATGTCTGTATACCGTCGACCTCTAGCTAGAGCTTGGCGTAATCATGGTCATAGCTGTTT
CCTGTGTGAAATTGTTATCCGCTCACAATTCCACACAACATACGAGCCGGAAGCATAAAGTGTAAAGCCTGGGGTGCCTA
ATGAGTGAGCTAACTCACATTAATTGCGTTGCGCTCACTGCCCGCTTTCCAGTCGGGAAACCTGTCGTGCCAGCTGCATT
AATGAATCGGCCAACGCGCGGGGAGAGGCGGTTTGCGTATTGGGCGCTCTTCCGCTTCCTCGCTCACTGACTCGCTGCGC

TCGGTCGTTCGGCTGCGGCGAGCGGTATCAGCTCACTCAAAGGCGGTAATACGGTTATCCACAGAATCAGGGGATAACGC
AGGAAAGAACATGTGAGCAAAAGGCCAGCAAAAGGCCAGGAACCGTAAAAAGGCCGCGTTGCTGGCGTTTTTCCATAGGC
TCCGCCCCCCTGACGAGCATCACAAAAATCGACGCTCAAGTCAGAGGTGGCGAAACCCGACAGGACTATAAAGATACCAG
GCGTTTCCCCCTGGAAGCTCCCTCGTGCGCTCTCCTGTTCCGACCCTGCCGCTTACCGGATACCTGTCCGCCTTTCTCCC
TTCGGGAAGCGTGGCGCTTTCTCAATGCTCACGCTGTAGGTATCTCAGTTCGGTGTAGGTCGTTCGCTCCAAGCTGGGCT
GTGTGCACGAACCCCCCGTTCAGCCCGACCGCTGCGCCTTATCCGGTAACTATCGTCTTGAGTCCAACCCGGTAAGACAC
GACTTATCGCCACTGGCAGCAGCCACTGGTAACAGGATTAGCAGAGCGAGGTATGTAGGCGGTGCTACAGAGTTCTTGAA
GTGGTGGCCTAACTACGGCTACACTAGAAGGACAGTATTTGGTATCTGCGCTCTGCTGAAGCCAGTTACCTTCGGAAAAA
GAGTTGGTAGCTCTTGATCCGGCAAACAAACCACCGCTGGTAGCGGTGGTTTTTTTGTTTGCAAGCAGCAGATTACGCGC
AGAAAAAAAGGATCTCAAGAAGATCCTTTGATCTTTTCTACGGGGTCTGACGCTCAGTGGAACGAAAACTCACGTTAAGG
GATTTTGGTCATGAGATTATCAAAAAGGATCTTCACCTAGATCCTTTTAAATTAAAAATGAAGTTTTAAATCAATCTAAA
GTATATATGAGTAAACTTGGTCTGACAGTTACCAATGCTTAATCAGTGAGGCACCTATCTCAGCGATCTGTCTATTTCGT
TCATCCATAGTTGCCTGACTCCCCGTCGTGTAGATAACTACGATACGGGAGGGCTTACCATCTGGCCCCAGTGCTGCAAT
GATACCGCGAGACCCACGCTCACCGGCTCCAGATTTATCAGCAATAAACCAGCCAGCCGGAAGGGCCGAGCGCAGAAGTG
GTCCTGCAACTTTATCCGCCTCCATCCAGTCTATTAATTGTTGCCGGGAAGCTAGAGTAAGTAGTTCGCCAGTTAATAGT
TTGCGCAACGTTGTTGCCATTGCTACAGGCATCGTGGTGTCACGCTCGTCGTTTGGTATGGCTTCATTCAGCTCCGGTTC
CCAACGATCAAGGCGAGTTACATGATCCCCCATGTTGTGCAAAAAAGCGGTTAGCTCCTTCGGTCCTCCGATCGTTGTCA
GAAGTAAGTTGGCCGCAGTGTTATCACTCATGGTTATGGCAGCACTGCATAATTCTCTTACTGTCATGCCATCCGTAAGA
TGCTTTTCTGTGACTGGTGAGTACTCAACCAAGTCATTCTGAGAATAGTGTATGCGGCGACCGAGTTGCTCTTGCCCGGC
GTCAATACGGGATAATACCGCGCCACATAGCAGAACTTTAAAAGTGCTCATCATTGGAAAACGTTCTTCGGGGCGAAAAC
TCTCAAGGATCTTACCGCTGTTGAGATCCAGTTCGATGTAACCCACTCGTGCACCCAACTGATCTTCAGCATCTTTTACT
TTCACCAGCGTTTCTGGGTGAGCAAAAACAGGAAGGCAAAATGCCGCAAAAAAGGGAATAAGGGCGACACGGAAATGTTG
AATACTCATACTCTTCCTTTTTCAATATTATTGAAGCATTTATCAGGGTTATTGTCTCATGAGCGGATACATATTTGAAT
GTATTTAGAAAAATAAACAAATAGGGGTTCCGCGCACATTTCCCCGAAAAGTGCCACCTGACGTC
